# ASO Author Reflections: Organoid Model to Aid the Decision of Hyperthermic Intraperitoneal Chemotherapy for Colorectal Cancer

**DOI:** 10.1245/s10434-024-16555-4

**Published:** 2024-11-20

**Authors:** Duo Liu, Keli Yang, Jian Cai

**Affiliations:** 1 Department of Colorectal Surgery, Shenzhen Second People’s Hospital, First Affiliated Hospital of Shenzhen University, Medical Innovation Technology Transformation Center of Shenzhen Second People’s Hospital, Shenzhen University, Shenzhen, China; 2 Department of Colorectal Surgery, Guangdong Institute of Gastroenterology, Guangdong Provincial Key Laboratory of Colorectal and Pelvic Floor Diseases, Biomedical Innovation Center, The Sixth Affiliated Hospital, Sun Yat-Sen University, Guangzhou, China

## Past

Cytoreductive surgery (CRS) combined with hyperthermic intraperitoneal chemotherapy (HIPEC) currently is widely adopted as a treatment for peritoneal metastasis of colorectal cancer in many centers around the world. Nevertheless, consensus regarding the HIPEC regimen remains elusive. A study shows a minimum of 60 distinct HIPEC regimens, predominantly using oxaliplatin and mitomycin C.^1^ Current retrospective studies have generated conflicting outcomes from investigating the efficacy of oxaliplatin and mitomycin C in HIPEC for colorectal cancer.^2,3^ However, it is difficult to conduct prospective studies to compare the efficacy of HIPEC between different drugs. Exploration of the efficacy of different HIPEC regimens *in vitro* may inform clinical decision-making.

## Present

Tumor organoids as preclinical models for drug sensitivity screening have been gradually used to guide clinical individualized drug use.^4^ In the current study, tumor organoids obtained from 46 patients with colorectal cancer were cultured, and in vitro hyperthermic perfusion experiments were performed on 42 organoids using the following five different regimens: mitomycin C, mitomycin C combined with cisplatin, mitomycin C combined with 5-fluorouracil, oxaliplatin, and oxaliplatin combined with 5-fluorouracil. The in vitro hyperthermic perfusion demonstrates that the inhibition rate of mitomycin C, both alone and in combination with cisplatin, surpasses that of mitomycin C combined with 5-fluorouracil and oxaliplatin.^5^

## Future

In clinical practice, the combination of mitomycin C and cisplatin can be regarded as the optimal choice for HIPEC in colorectal cancer. Oxaliplatin may not be the first choice for HIPEC for colorectal cancer, and further studies are needed to confirm the mechanism of oxaliplatin resistance in HIPEC for colorectal cancer. As a preclinical model, tumor organoids can be used as a reference for more medical decisions.

## References

[1] Yurttas C, Hoffmann G, Tolios A, Haen SP, Schwab M, Königsrainer I, et al. Systematic review of variations in hyperthermic intraperitoneal chemotherapy (HIPEC) for peritoneal metastasis from colorectal cancer [J]. *J Clin Med.* 2018;7:567. 30572653 10.3390/jcm7120567 PMC6306814

[2] Prada-Villaverde A, Esquivel J, Lowy AM, Markman M, Chua T, Pelz J, et al. The American society of peritoneal surface malignancies evaluation of HIPEC with mitomycin C versus oxaliplatin in 539 patients with colon cancer undergoing a complete cytoreductive surgery [J]. *J Surg Oncol*. 2014;110:779–85. 25088304 10.1002/jso.23728

[3] Leung V, Huo YR, Liauw W, Morris DL. Oxaliplatin versus mitomycin C for HIPEC in colorectal cancer peritoneal carcinomatosis [J]. *Eur J Surg Oncol*. 2017;43:144–9. 27780675 10.1016/j.ejso.2016.09.015

[4] Wensink GE, Elias SG, Mullenders J, Koopman M, Boj SF, Kranenburg OW, et al. Patient-derived organoids as a predictive biomarker for treatment response in cancer patients [J]. *NPJ Precision Oncol*. 2021;5:30. 10.1038/s41698-021-00168-1 PMC8042051 33846504

[5] Liu D, Chen ZX, Deng WH, et al. An organoid model for the therapeutic effect of hyperthermic intraperitoneal chemotherapy for colorectal cancer. *Ann Surg Oncol.* 2024. 10.1245/s10434-024-16469-1. 10.1245/s10434-024-16469-1 PMC11811434 39589577

